# Star Polymers as Non-Viral Carriers for Apoptosis Induction

**DOI:** 10.3390/biom12050608

**Published:** 2022-04-19

**Authors:** Agnieszka Fus-Kujawa, Łukasz Sieroń, Estera Dobrzyńska, Łukasz Chajec, Barbara Mendrek, Natalia Jarosz, Łukasz Głowacki, Kamila Dubaj, Wojciech Dubaj, Agnieszka Kowalczuk, Karolina Bajdak-Rusinek

**Affiliations:** 1 Department of Medical Genetics, Faculty of Medical Sciences in Katowice, Medical University of Silesia, Katowice, Medykow 18 Street, 40-752 Katowice, Poland; afus@sum.edu.pl (A.F.-K.); lukasz.sieron@sum.edu.pl (Ł.S.); edobrzynska@sum.edu.pl (E.D.); natalia.jarosz@sum.edu.pl (N.J.); biolmolgen@sum.edu.pl (Ł.G.); s73151@365.sum.edu.pl (K.D.); s74939@365.sum.edu.pl (W.D.); 2 Animal Histology and Embryology Group, Institute of Biology, Biotechnology and Environmental Protection, Faculty of Natural Sciences, University of Silesia in Katowice, 40-007 Katowice, Poland; lukasz.chajec@us.edu.pl; 3 Centre of Polymer and Carbon Materials, Polish Academy of Sciences, M. Curie-Sklodowskiej 34, 41-819 Zabrze, Poland; bmendrek@cmpw-pan.edu.pl (B.M.); akowalczuk@cmpw-pan.edu.pl (A.K.); 4 Students Scientific Society, Faculty of Medical Sciences in Katowice, Medical University of Silesia, 40-752 Katowice, Poland

**Keywords:** apoptosis, star polymers vectors, delivery systems, cross-membrane transport

## Abstract

Apoptosis is a widely controlled, programmed cell death, defects in which are the source of various diseases such as neurodegenerative diseases as well as cancer. The use of apoptosis in the therapy of various human diseases is of increasing interest, and the analysis of the factors involved in its regulation is valuable in designing specific carriers capable of targeting cell death. Highly efficient and precisely controlled delivery of genetic material by low-toxic carriers is one of the most important challenges of apoptosis-based gene therapy. In this work, we investigate the effect of the star polymer with 28 poly(*N,N*′-dimethylaminoethyl methacrylate) arms (STAR) on human cells, according to its concentration and structure. We show that star polymer cytotoxicity increases within its concentration and time of cells treatment. Except for cytotoxic effect, we observe morphological changes such as a shrinkage, loss of shape and begin to detach. We also prove DNA condensation after star polymer treatment, one of the most characteristic feature of apoptosis. The results indicate that the use of STAR triggers apoptosis in cancer cells compared to various normal cells, what makes these nanoparticles a promising drug in therapeutic strategy, which targets apoptosis. We demonstrate highlighting potential of star polymers as an innovative tool for anti-cancer therapy.

## 1. Introduction

Apoptosis is a form of programmed cell death, during which morphological and biochemical changes occur, including permeabilization of the outer mitochondrial membrane, cytoplasmic condensation, nuclear fragmentation, and rearrangements resulting in generation of apoptotic bodies. This cell death type is finely regulated at gene level resulting in the orderly and efficient removal of damaged cells such as those occurring after DNA damage or during development [1,2]. The machinery of apoptosis is complex and involves many signaling pathways [1]. It can be triggered in a cell through either the caspase-mediated extrinsic or intrinsic pathways. Both pathways converge to activate the effector apoptotic caspases resulting ultimately in morphological and biochemical cellular alterations, characteristics for apoptosis [3,4]. Usually, the balance between the proapoptotic and antiapoptotic protein regulators is a critical key point to determine if a cell undergoes apoptosis. The induction of apoptosis as a result of DNA damage in precancerous lesions can remove potentially harmful cells, thereby blocking tumor growth. Impaired elimination of damaged cells by apoptosis is associated with autoimmune diseases, whereas excessive apoptosis induces immunological tolerance and reduces inflammatory responses. Defects in apoptosis are the source of various diseases such as neurodegenerative, infectious, heart diseases and also cancer [5].

Since application of apoptosis has been evident for therapy of various human diseases, it has gained importance for basic research [6]. During the last few decades, prominent progress has been achieved in the field of therapeutics based on the phenomenon of apoptosis. Nowadays, apoptosis-targeting therapies are advancing from preclinical/clinical trials to actual application.

Highly efficient and precisely controlled delivery of genetic material by carriers featured with their low toxicity is one of the most important challenge faced by apoptosis-based gene therapy [7,8,9]. In most cases, the therapeutic systems are based on the use of nanoparticles involving polymeric and inorganic carriers or their combination. These nanoparticles are of great interest due to target delivery, their limited size and increased delivery efficiency [10,11]. Some nanoparticles may display a toxic effect but do not induce apoptosis via targeting the overexpressed anti-apoptotic proteins or stimulation of the expression of pro-apoptotic molecules [12]. The other important challenge is to find a versatile carrier that allows to target apoptosis for specific cell types and not for all cell types. Although viral vectors are effective, they have numerous disadvantages including activation of the immune response that may cause clinical implications and decreases therapy efficiency. Therefore, it is still a need for searching synthetic, non-immunogenic delivery systems that exhibit low cytotoxicity as well as high fidelity. DQAsomes (dequalinium-based liposome-like vesicles) have been also analyzed as the prototype for all mitochondria-targeted vesicular pharmaceutical nanocarrier systems. It has been proven that there is a higher transfection efficiency with star polymers compared with cationic lipids [13].

Among the variety of delivery vectors with a promising perspective are synthetic star polymers.

Star polymers are branched macromolecules in which linear polymer chains are covalently bound to central element “the core”. For many years, the work that has been put in the field of polymeric stars has focused more on controlling the structure of stars than on their biofunctions. In recent years, due to the relative ease of synthesis, possible high molar masses, multifunctionality, and therefore, unique intrinsic properties, they are of significant interest for gene delivery applications. Until now, many kinds of star polymers with polycationic arms have been synthetized and used for gene transfection [14,15,16,17]. The main factor limiting the transfection efficiency is the toxicity of star polycations. However, the influence of their chemical structure, as well as molar mass on toxicity for treated cells has been revealed [16,18].

In this article, we investigate the effect of star polymers with 28 poly(*N,N*′-dimethylaminoethyl methacrylate) arms (STAR) and their action on treated cells. Our idea of using star polymers as nanoparticles inducing apoptosis in treated cells gives new insight to anti-cancer mechanisms. Maintenance of the properly functioning apoptotic pathways is valid to provide a relevant balance between cell death and cell survival and preserve genome integrity. It should be expected that the chemical structure of star polymers strongly affects induction of apoptosis. Therefore, cationic polymers as nanoparticles with a different range of cytotoxicity and the ability to induce cell death can be considered as a potential candidate for anticancer drugs.

## 2. Materials and Methods

### 2.1. Cell Culture

Human fibrosarcoma cells HT-1080 (ATCC#CCL-121), human dermal fibroblasts PDF (ATCC#CRL-2522), human bladder cancer cell lines 5637 (ATCC# HTB-9) and T24 (ATCC#HTB-4), Primary Mammary Epithelial Cells (HMEC) (PCS-600-010™) and Primary Bladder Epithelial Cells (BdEC) (PCS-420-010™) were purchased from the American Type Culture Collection (ATCC, Manassas, VI, USA). The HT-1080 and PDF cells were cultured in a cell culture medium consisting of Dulbecco’s Modified Eagles Medium (DMEM) (Sigma aldrich, Darmstadt, Germany) supplemented with 10% FBS, 1% L-glutamine, 1000 U/mL of penicillin, 100 µg/mL of streptomycin and 250 µg/mL of amphotericin B at 37 °C and 5% CO_2_. T24 and 5637 cells were cultured in McCoy’s 5a Medium Modified (Sigma aldrich, Darmstadt, Germany), and RPMI-1640 Medium (Sigma aldrich, Darmstadt, Germany), respectively, and supplemented with 10% FBS at 37 °C and 5% CO_2_. HMEC cells were cultured in Mammary Epithelial Cell Basal Medium supplemented with the Mammary Epithelial Cell Growth Kit (ATCC, Manassas, VI, USA) at 37 °C and 5% CO_2_. BdEC cells were cultured in Bladder Epithelial Cell Basal Medium supplemented with Bladder Epithelial Growth Kit (ATCC, Manassas, VI, USA) at 37 °C and 5% CO_2_.

### 2.2. Synthesis of Star Polymer with Poly(Arylene Oxindole) Core and Poly(N,N′-Dimethylaminoethyl Methacrylate) Arms

Star polymers with 28 poly(*N,N*′-dimethylaminoethyl methacrylate) arms (STAR) were obtained by the “core first” method via controlled atom transfer radical polymerization (ATRP) as previously described [16] yielding polymer of Mn = 370,000 Da and dispersity index (Mw/Mn) equal to 1.54.

Zeta potential measurements were performed on a Zetasizer Nano ZS 90 (Malvern Instruments) in disposable folded capillary cells in triplicate (Supplementary Materials; Table S1).

### 2.3. Cytotoxicity Assay

HT-1080 cells were seeded in 24-well plates at a density of 8 × 10^4^ per well. The following day, the cell culture medium was supplemented with tested star polymer to the desired final concentrations: 0 (control) 5, 10, 20, 30, 40 and 50 µg/mL. Cells were incubated with the polymer for 2, 4, 6, 8 and 24 h. After incubation time, cells were washed with pre-warmed phosphate-buffered saline (PBS, Sigma aldrich, Darmstadt, Germany), subsequently 200 µL of pre-warmed Alamar blue reagent in cell culture medium was added to a final concentration of 10%. Cells were incubated at 37 °C and 5% CO_2_ for 1 h. Subsequently, 100 µL of the mixture was transferred to a new well of 96-well TPP plate (PerkinElmer, Waltham, MA, USA) and the fluorescence emission was monitored at 590 nm using a VICTOR^TM^ Multilabel Plate Reader (PerkinElmer, Waltham, MA, USA) with a 560 nm excitation source. The cell viability was assessed based on the percentage of live cells compared to the untreated control cells. For 5637, T24, human dermal fibroblasts, Primary Mammary Epithelial Cells and Primary Bladder Epithelial Cells analysis were performed according to the same protocol. Cells were treated with star polymer for 6 and 24 h.

### 2.4. Fluorescence

Cells were stained with 300 nM of DAPI solution (2-[4-(Aminoiminomethyl)phenyl]-1H-Indole-6-carboximidamide hydrochloride) in the dark and visualized under an inverted light microscope. In order to achieve apoptosis/necrosis visualization, cells were stained with 0.2 µg/mL of acridine orange (AO; Sigma Aldrich, Darmstadt, Germany) and 5 µg/mL of ethidium bromide (EtBr; Sigma Aldrich, Darmstadt, Germany) and observed under an inverted light microscope (OLYMPUS, Poland).

### 2.5. Apoptosis Assay

Star polymer was added to HT-1080 cells. After 6 h, cells were stained with annexin V and propidium iodide using an annexin V Apoptosis Detection Kit FITC (Thermofisher, Germany) according to manufacturer’s protocol. Annexin V and propidium iodide (PI) were detected using FITC and PE channel, respectively.

### 2.6. Flow Cytometry

For flow cytometry analysis cells were treated with star polymer for 6 h. Subsequently, membrane potential was assessed using the potentiometric dye tetramethyl rhodamine methyl ester (TMRM; Sigma Aldrich, Darmstadt, Germany) at a final concentration of 100 µM for 10 min at 37 °C. As a positive control for mitochondrial depolarization we used CCCP (carbonyl cyanide 3-chlorophenylhydrazone) (Sigma Aldrich, Darmstadt, Germany) at a final concentration of 10 µM at 37 °C for 20 min. Intracellular reactive oxygen species (ROS) were detected using 7 µM of 2’7’-dichlorodihydrofluorescein diacetate (DC-FDA; Sigma aldrich, Darmstadt, Germany). Cells were incubated for 15 min at room temperature with DC-FDA and immediately visualized. Cells were treated with 1 mM of H_2_O_2_ for 4 h was used as a positive control for ROS production. Cell analysis was performed with a FACS Aria instrument (BD Biosciences, San Jose, CA, USA). DC-FDA was detected on FITC channel and TMRM on PerCP-Cy5-5 channel. In order to assess DNA content in HT-1080 cells, cells were fixed with 70% ethanol (EtOH) and proceeded according to methods described by Riccardi and Nicoletti [19]. Stained cells were analyzed using PE channel.

### 2.7. RNA Isolation and Quantitative RT-PCR

Total RNA was isolated using the RNeasy Plus Mini Kit (Qiagen, Germany) according to the manufacturer’s protocol. cDNA was synthesized from 2 µg RNA with the Revert Aid First Strand cDNA Synthesis Kit (Thermo Scientific, Germany) according to the manufacturer’s instructions. Relative expression levels were measured in triplicates in a Roche Light Cycler 480 using Power SYBR Green PCR Master Mix (Applied Biosystems, Germany), 300 mM primers (Supplementary Materials; Table S2) and 1/15 cDNA stock. Values were calculated using the Pfaffl method and normalized to those of GAPDH.

### 2.8. Statistical Analysis

Statistical analyses of the qRT-PCR data were performed with Microsoft Excel software. Normalized relative expression levels were used to calculate the mean and the SEM of all experiments (represented by columns and error bars in the figures, *n* = 3). The two-tailed student’s *t* test was used to assess statistical significances. In all figures, p-values of statistical significance are represented as follows: * *p* < 0.05; ** *p* < 0.01; *** *p* < 0.001; **** *p* < 0.0001.

## 3. Results

### 3.1. Toxicity of Star Polymer Is Concentration-Dependent and Induces Mainly Early Apoptosis in HT-1080 Cells 

We tested kinetics of star polymer action on HT-1080 fibrosarcoma cells based on their cytotoxicity. It has been analyzed before that the structure of the star polymer has a significant impact on its cytotoxic effect [18]. Although star polymers are often analyzed as nanoparticles for the transport of numerous bioactive substances [20,21], not enough attention has been devoted to their action as apoptosis or necrosis inducers.

Star macromolecules, used as potential drug/gene delivery vehicles should ensure good solubility of active substances in aqueous media and protect the substances molecules of interest from undesirable inactivation and decomposition. Polymers are uptaken by endocytosis, what results in their localization in the endosome. Interactions of the amine groups of cationic polymers with plasma membrane affect the membrane integrity and lead to the passive influx of water into the cell. Passive diffusion of polymers into the cells can disrupt the integrity of other cellular organelles as a result of their membranes’ perforation.

**Figure 1:**
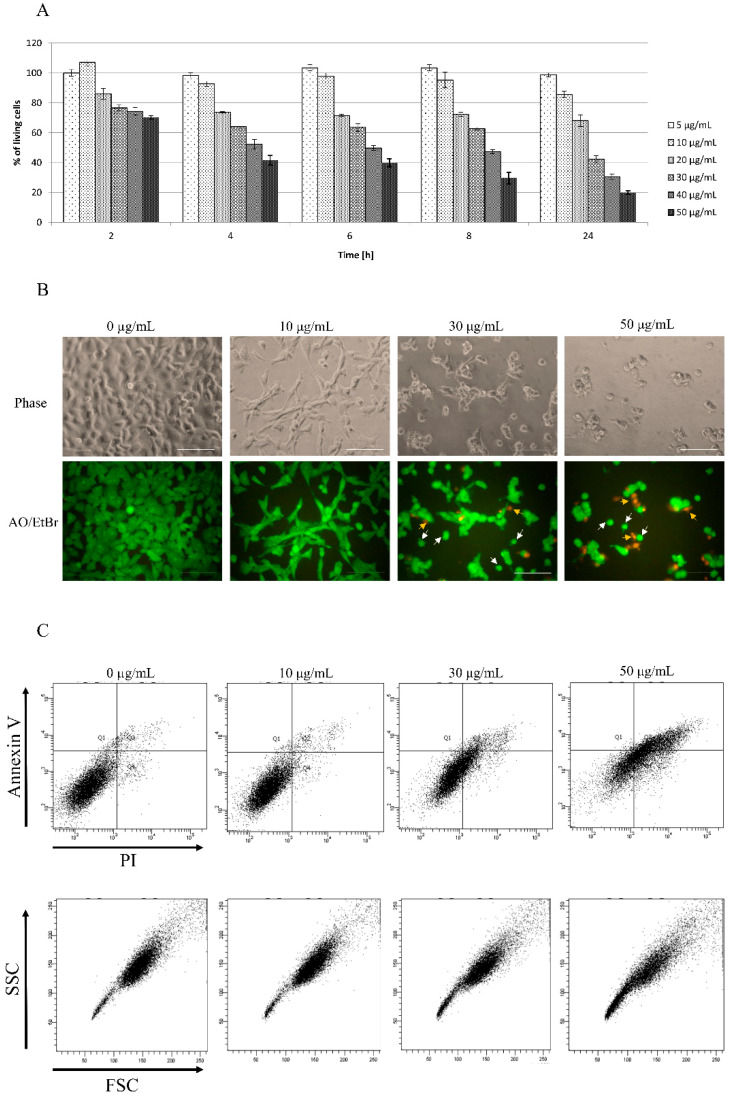
Kinetics of star polymer action on HT-1080 cells. (**A**) The cytotoxicity assay of the tested star polymer. The assay was performed with HT-1080 cells. The results are presented as a % of cells surviving in the presence of the star polymer in the range of concentrations (5–50 µg/mL). (**B**) HT-1080 cells after 6 h of treatment with star polymer at indicated concentrations were seen under light microscope (upper panel) and under fluorescence microscope (lower panel) following nuclear staining with acridine orange/ethidium bromide (AO/EtBr) double staining. Cells with features of early apoptosis are indicated by white arrows and late apoptosis by yellow arrows. Scale bars represent 100 µm. (**C**) Flow cytometry analysis of cell death type in HT-1080 cells treated with star polymer after 6 h using annexin V and PI staining.

Despite star polymers as non-viral carriers being less toxic compared to viral carriers, higher concentrations may be lethal for treated cells. The crucial aim was to find the optimal concentration of star polymer with precisely matched chemical structure. We observed the increase of star polymer cytotoxicity within its concentration and time of cell treatment. In cells treated for 6 h with 30 µg/mL of star polymer, we observed a cytotoxic effect with 60% of cell viability. At a concentration of 50 µg/mL, we found only 20% of living cells after 24 h (Figure 1A).

A cytotoxicity assay has been also confirmed by HT-1080 cells morphology visualization after 6 h of treatment with star polymer (Figure 1B, upper line). Cells undergo morphological changes such a shrinkage, loss of shape and beginning to detach. In control cells, we did not observe signs of either apoptosis nor necrosis (Figure 1B, upper line). Distinguishing cell death types under a microscope may not to be obvious thus double staining with acridine orange (AO) and ethidium bromide (EtBr) has been performed (Figure 1B, lower panel). Early apoptotic cells are showed as bright green or yellow with a rounded shape whereas late apoptotic cells are red with condensed or fragmented nuclei. Moreover, necrotic cells appear red with normal nucleus and wide shape. Cells with features of early apoptosis are indicated by white arrows and of late apoptosis by yellow arrows (Figure 1B, lower panel). Most of apoptotic cells appeared as early apoptotic. Necrotic cells were rare and were detected only at higher polymer concentrations (50 µg/mL) (Figure 1B, lower panel).

In terms of biochemistry, the presence of phosphatidylserine on outer surface of cells, detected by annexin V binding, is a marker of apoptotic cells, whereas propidium iodide (PI) is a marker of dead cells with disrupted integrity of the plasma membrane. Viable cells are negative for both markers whereas early apoptotic are positive for annexin V and negative for PI. Late apoptotic cells are positive for both markers and necrotic cells are positive only for PI. According to cytotoxicity dynamics and changes in morphology of HT-1080 cells, we chose 6 h of treatment with star polymer as the proper time for annexin V and PI staining analysis. Control cells mostly remained negative for annexin V and PI whereas apoptotic and necrotic cells consisted in only an insignificant fraction of analyzed cells. Addition of the star polymer into the cell culture increased the staining with both markers, annexin V and PI, simultaneously (Figure 1C, upper panel). The intensity of the staining is positively correlated with star polymer concentration. The shift in stained cells applies to all cell populations as the entire population goes directly to late apoptosis, omitting the late phase. Additionally, the analysis of cells by their size and granularity, depicted by forward scatter (FSC) and side scatter (SSC), respectively, revealed an increased number of more complex cells proportional to the concentration of star polymer (Figure 1C, lower panel).

**Figure 2:**
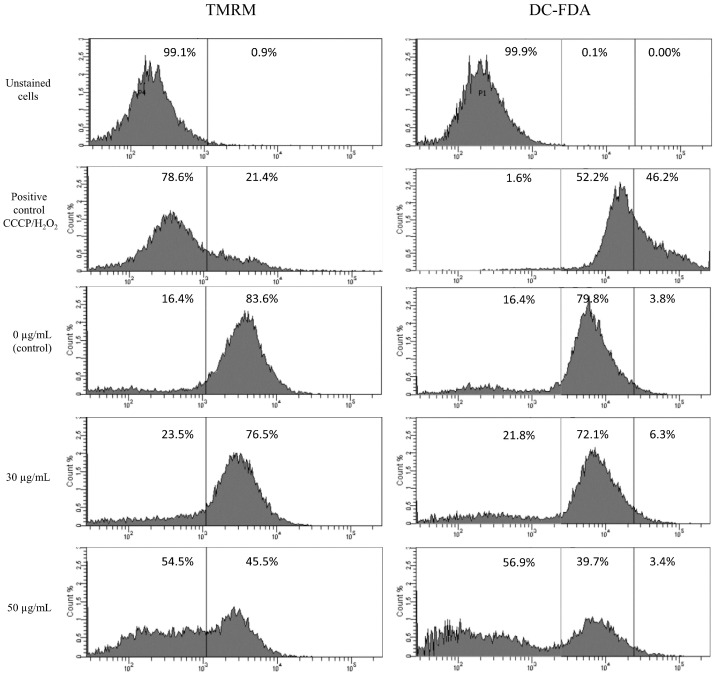
Flow cytometry analysis of mitochondrial potential (TMRM staining) and ROS production (DC-FDA) in HT-1080 cells after 6 h of treatment with star polymer at indicated concentrations. CCCP (**left panel**) and H_2_O_2_ (**right panel**) as positive controls for mitochondrial depolarization and ROS production, respectively. Percentage of positive cells are presented on each histogram.

The main pathways of apoptosis are the extrinsic and intrinsic as well as a perforin/granzyme pathway. Each pathway requires specific signals to begin a cascade of molecular events. We analyzed changes in mitochondrial potential using staining with TMRM and ROS production using DC-FDA staining in HT-1080 cells after 6 h of star polymer treatment (Figure 2). We used unstained cells as a reference for cell population location. Control cells were not treated with star polymer and showed a presence of mitochondrial potential reflected by positive staining with TMRM. Additionally, we used CCCP and H_2_O_2_ as positive controls for mitochondrial depolarization and ROS production, respectively. After 6 h of cell treatment with star polymer at a concentration of up to 30 µg/mL, only a slight change in the number of stained cells was observed. A significant shift occurred at the higher polymer concentration (50 µg/mL). A similar effect was observed with DC-FDA, that is a common marker of ROS levels.

**Figure 3:**
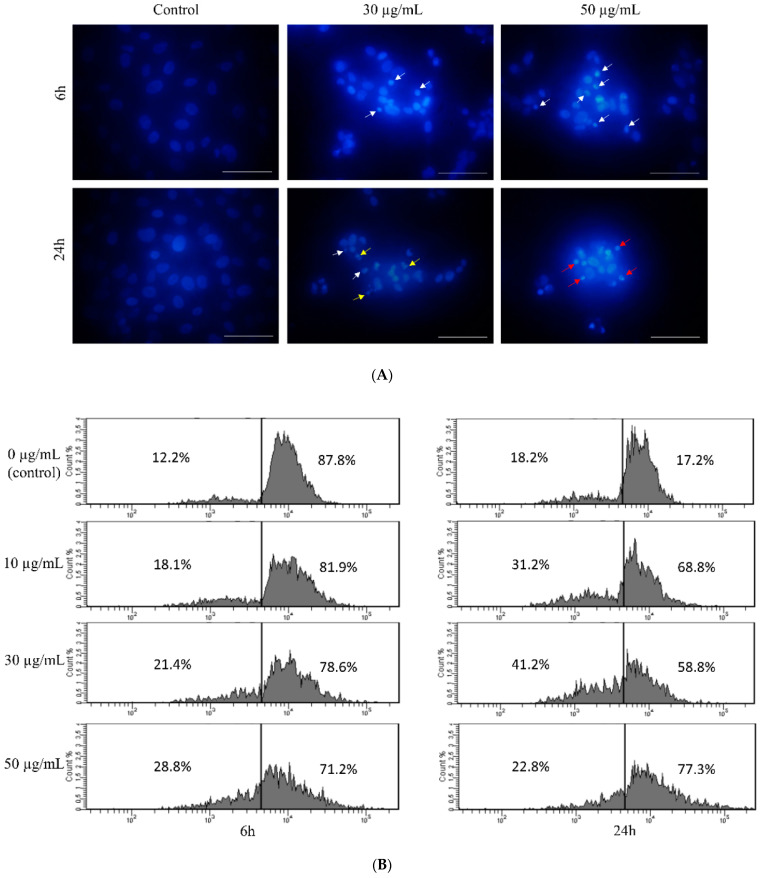
Changes in nuclear morphology of HT-1080 cells after treatment with star polymer. (**A**) DAPI-stained nuclei of HT-1080 cells after treatment with star polymer. In untreated cells, nuclei are large and chromatin is uncondensed. In treated cells shrinkage of nucleus and chromatin condensation are observed. Nuclei with condensed chromatin (early stage of apoptosis) is indicated by white arrows. Fragmented nuclei are indicated by yellow arrows and nuclei with a ring-like shape are indicated by red arrows. Scale bar represents 50 µm. (**B**) Flow cytometry analysis of DNA content in HT-1080 cells treated with star polymer for 6 h (left panel) and 24 h (right panel). Percentage of positive cells are presented on each histogram.

Although the ROS level remains unchanged at a concentration of 30 µg/mL, we performed nuclei visualization with DAPI in order to detect any changes in genetic material. Observation of DAPI-stained nuclei of HT-1080 cells revealed that nuclei of control cells present normal morphology after 6 and 24 h of treatment with star polymer (Figure 3A). Nevertheless, shrunken nuclei are presented in cells treated with star polymer (indicated by arrows). The number of cells with a condensed nucleus were in positive correlation with star polymer concentration. Importantly, after 6 h of treatment, we did not observe fragmented nuclei that are typical for apoptotic bodies formation. After 24 h, the number of cells with condensed nuclei was increased. However, at higher concentrations (50 µg/mL) of star polymer, the shrunken nuclei remained unfragmented but with condensed chromatin localized under the nuclear envelope with a ring-like pattern (Figure 3A). Moreover, flow cytometry analysis of HT-1080 cells treated with star polymer for 6 and 24 h showed a decreased amount of DNA in HT-1080 cells, the size of which corresponds to the polymer concentration and incubation time (Figure 3B). In general, after 24 h of star polymer treatment, the population of DNA-deficient cells was significantly higher than after 6 h of treatment.

Cytotoxicity of star polymer on HT-1080 cells is concentration-dependent and induces morphological changes of treated cells. Cells double stained with OA and EtBr are mostly early apoptotic cells. Apart from these features that are characteristic for the apoptotic cells, abnormalities should also occur at molecular level. Accordingly, based on the previous results, we continued with analysis of apoptotic cells molecular profile.

### 3.2. HT-1080 Cells Treatment with Star Polymer Triggers Changes of Gene Expression Profile and Activates Various Apoptotic Pathways

**Figure 4:**
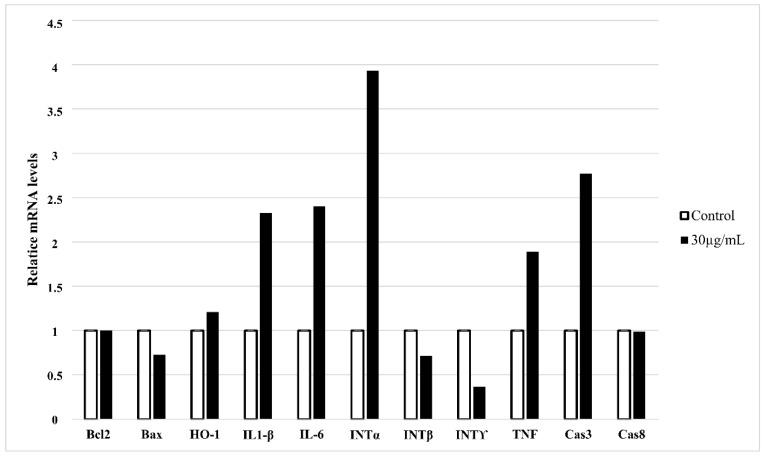
Quantitative RT-PCR expression analysis of apoptosis-related and inflammatory-related genes in HT-1080 cells treated with star polymer for 6 h at the concentration 30 µg/mL. GAPDH was used as loading control. Values are mean ± SEM (*n* = 3).

Except for morphological and biochemical changes that occur in cells undergoing apoptosis, the profile of gene expression also shows specific changes. Based on the gene expression profile, it is possible to assess the type of apoptotic pathway. We checked the expression of apoptosis-involved genes and those specific for inflammation in HT-1080 cells treated with 30 µg/mL of star polymer for 6 h (Figure 4). We did not observe changes in expression of BCL-2 and BAX genes involved in mitochondrial apoptotic pathway as well as for caspase 8 involved in external apoptotic pathway. However, we detected a slight increase in the expression of effector caspase 3. It corresponds to the DNA fragmentation that we observed in the cells treated with the star polymer.

Heme oxygenase (HO-1) plays an antiapoptotic and cytoprotective role in cells during stress-induced apoptosis. We observed expression of HO-1 only slightly increased (Figure 4). However, it is widely known that introduction of foreign carriers into cells may be stressful factor for treated cells. We also noticed increased expression of proinflammatory genes as interleukin 1β (IL-1β), interleukin 6 (IL-6) and TNF. Among interpherones, only expression of interpherone α (INT α) was elevated, whereas expression of other interpherones: β (INT β) and γ (INT γ) was decreased. It may be a result of different apoptotic pathways that are activated after cell treatment with star polymer in a cell-line-dependent manner [22].

### 3.3. Star Polymer Causes the Early Apoptosis in Human Bladder Cancer Cell Lines

**Figure 5:**
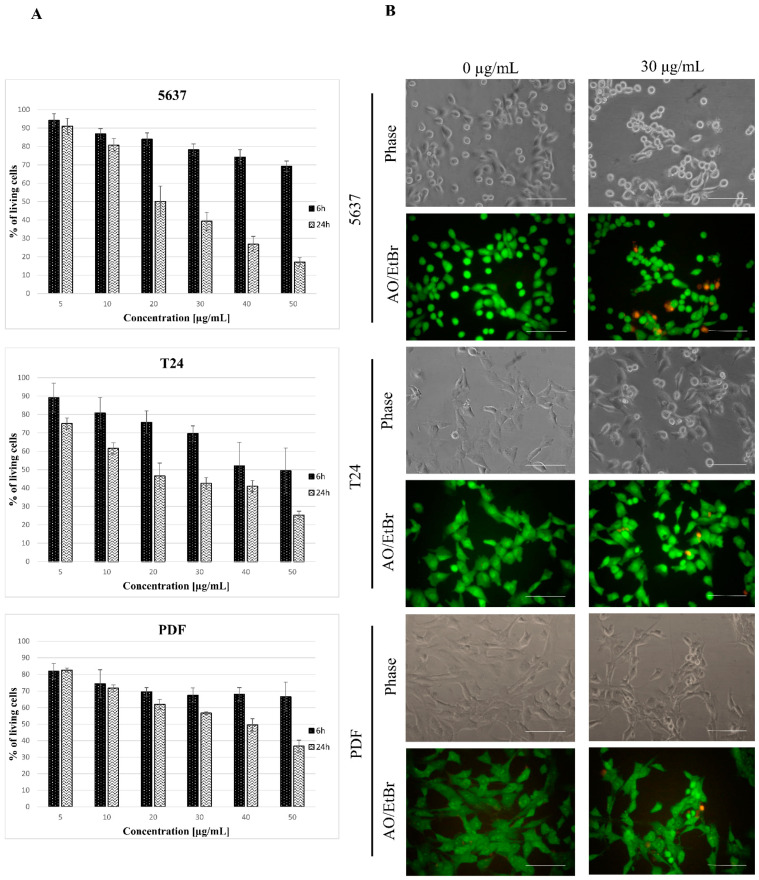
Kinetics of star polymer action on human bladder cancer cell lines and human dermal fibroblasts (PDF). (**A**) Cytotoxicity dynamics of star polymer for 5637 and T24 cancer cell lines and PDF after 6 and 24 h of treatment. (**B**) Cells’ morphology after 6 h of star polymer treatment at the concentration 30 µg/mL. Cells were double stained with acridine orange/ethidium bromide (AO/EtBr) in order to distinguish the cell death type. Scale bars represent 100 µm.

To check whether tested star polymer can serve as apoptotic inductor for other cell lines, we used human bladder cancer cell lines 5637 and T24, human dermal fibroblasts (PDF), Primary Mammary Epithelial Cells (HMECs) and Primary Bladder Epithelial Cells (BdECs) as additional cellular models. A cytotoxicity assay was performed after 6 and 24 h as at these time points, the most changes have been observed in HT-1080 cells. It has been revealed that the analyzed star polymer shows a toxic effect for tested cells in a concentration-dependent manner (Figure 5A). The level of toxicity varies between cell lines. The 5637 cells appeared to be the most sensitive, but the effect of star polymer treatment was clearly visible after 24 h compared to 6 h of treatment. Similarly, in T24 cells, a significant reduction in cell viability was also observed after 24 h. Among various normal cell lines, the most sensitive cells were PDF cells. In BdECs cells only slight toxic effect of STAR was observed (Supplementary Materials, Figure S1). However, in all cell lines, we observed the tendency of a reduction in cell viability that progressed over time.

**Figure 6:**
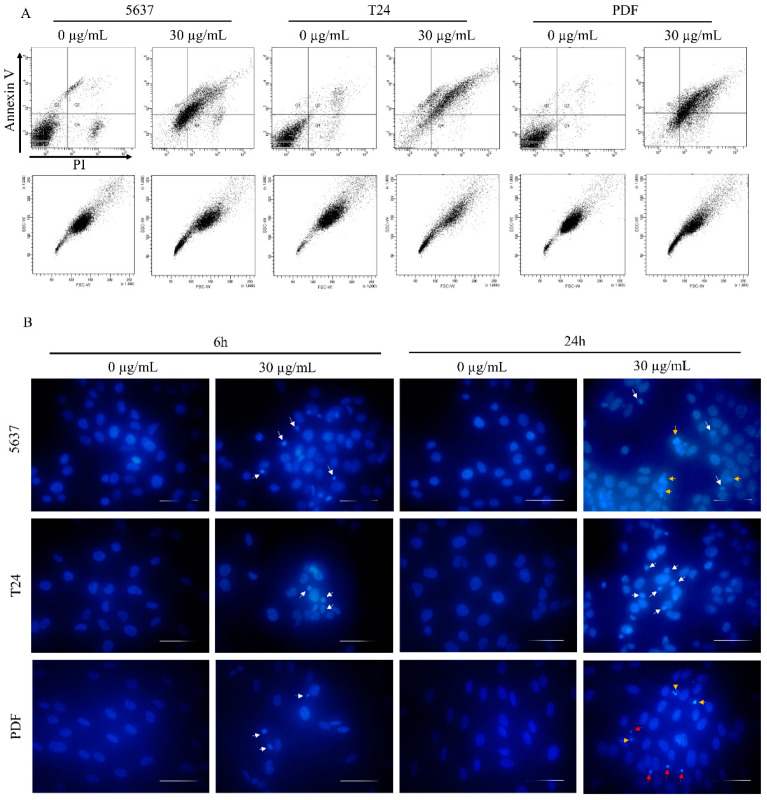
Cell death type assessment. (**A**) Flow cytometry analysis of 5637, T24 and PDF cells treated with star polymer for 6 h using Annexin V and Propidium Iodide staining. (**B**) DAPI-stained nuclei of 5637, T24 and PDF cells after treatment with star polymer for 6 h at the concentration 30 µg/mL. Condensed nuclei are indicated by white arrows, fragmented by yellow arrows and highly condensed nuclei by red arrows. Scale bars represent 50 µm.

The 5637 cells’ morphology was similar to that observed for HT-1080 cells, and they were more shrunken than the control cells (Figure 5B). AO/EtBr double staining showed the presence of bright green/yellow stained early apoptotic cells and a small number of dead cells with features of late apoptosis. Evaluation of cell death type by annexin V and PI staining revealed a similar pattern as for HT-1080 cells in all tested cell lines (Figure 6A). Analysis of the nuclei in cells treated with a star polymer at a concentration of 30 µg/mL, after 6 and 24 h of polymer treatment, revealed the presence of shrunken nuclei with condensed chromatin (Figure 6B). After 6 h of treatment in all cell lines, among normal nuclei also those with early stages of chromatin condensation were visible. After 24 h of incubation, condensed nuclei were more frequent in all cell lines. In PDF cells, the nuclei contained condensed chromatin divided or not divided. Similarly, fragmented nuclei were visible in 5637 cells. In T24 cells, the nuclei of dead cells stayed at early stages of condensation.

We demonstrated action of analyzed star polymer in various cell lines. The presence of early apoptotic cells has been confirmed as well. Morphological changes observed in 5637, T24 and PDF cells were similar to those detected in HT-1080 cells.

## 4. Discussion

Cancer is the second leading cause of death globally and despite the great effort has been done by researchers, its treatment still remains inefficient. The most conventional option is chemotherapy, but it can lead to severe adverse effects such as cell death of healthy tissues as well as the cancerous ones [23].

Cells undergoing reprogrammed cell death called apoptosis show some distinctive features such as cell shrinkage and blebbing. Cells undergo condensation and fragmentation of chromatin and nucleosomal DNA in order to form apoptotic bodies that are small vesicles. Generated apoptotic bodies are engulfed by phagocytes. The occurrence of apoptosis guarantees efficient removal of impaired and damaged cells, which is important for maintaining proper cell proliferation and preventing the development of cancer. Defects or insufficient amounts of apoptotic processes can lead to uncontrolled cell proliferation. In contrast, excessive apoptosis is associated with atrophy or ischemic heart disease [5]. The biological mechanism of apoptosis is exceedingly sophisticated. It is a precisely controlled process, although the molecular mechanism is still unclear. Experiments have revealed that abnormalities in cells death can lead to many diseases. Taking it into account, analysis of the factors involved in the regulation of apoptosis is valuable in the designing of specific carriers that are able to target cell death [5,24].

Although apoptosis is precisely controlled process, many factors are known to be inducers of this cell death type. Until now, folate modified liposomes loaded with bleomycin, modified chitosan nanoparticles or methotrexate were investigated as a potent anticancer system. These nanoparticles induce anticancer activity in a time- and dose-dependent manner but they still demonstrate selective cellular uptake [25,26,27].

Herein, we analyzed the action of star polymer with 28 poly(*N,N*′-dimethylaminoethyl methacrylate) arms (STAR). The cytotoxicity of linear poly(*N,N*′-dimethylaminoethyl methacrylate) has been previously described for HepG2 cells, showing that cellular membrane disruption and polymer interactions with negatively charged proteins increase with the molar mass of polycation [16,28]. Polycationic nanoparticles are widely discussed as non-viral carriers of genetic material. However, the comprehensive analysis is also required before they will be used for pre-clinical studies. Nevertheless, the molecular mechanism of action of these polymers is still unclear. Cytotoxicity assay of HT-1080 cells as model cells showed that toxicity of analyzed star is concentration-dependent (Figure 1A). Except for cytotoxic effect that occurs since a concentration of 30 µg/mL, we also observed morphological changes in treated cells (Figure 1B). It is in an agreement with other experiments that revealed the toxic effect of non-viral carriers at higher concentrations [28]. Moreover, analysis of live and dead cells showed that dead cells start to appear at a concentration of 30 µg/mL (Figure 1B). It is commonly known that cells undergo a characteristic shrinkage at the early stage of apoptosis. They are smaller while the content within the cell is highly packed. In late apoptosis, nucleus fragmentation occurs. It is accompanied by further plasma membrane blebbing and formation of small apoptotic bodies. Nowadays, it is known that apoptotic bodies contain compounds of cells such as cytoplasm, organelles, and nuclear content. These bodies are then engulfed by phagocytes for final degradation. The final result of apoptosis is phagocytosis which prevents spillage of hazardous materials packed within the apoptotic cells into the surroundings. The reason for forming apoptotic bodies remains vague.

We assessed the type of death that occurs in cells treated with the analyzed star polymer and used annexin V and PI assay that is the common test for this purpose. We have shown that cells treated with star polymer die apoptotic (Figure 1C) and the level of ROS in these cells were slightly changed at a concentration of 30 µg/mL (Figure 2). Importantly, it is well known that an excess of antioxidant results in dramatic consequences [29] as well as prooxidant excess determine the oxidative stress such as aging and death [30].

Although apoptotic pathways are widely described in literature [31,32,33,34,35] many aspects need to be considered in characterizing this type of cell death. Cationic substances are driven into mitochondria by the potential of mitochondrial membrane and this property can be used for intramitochondrial transport of drugs. The mitochondrial pathway may be activated in response to cellular stresses, including mitochondrial DNA (mtDNA) damage, heat shock, hypoxia or endoplasmic reticulum stress [36,37].

Intracellular ROS are produced mostly in mitochondria, as a result of leakage from the respiratory electron transport chain. Importantly, ROS induces cell survival response at lower doses, while they are responsible for apoptosis activation at higher doses [38,39]. These radicals are supposed to activate the intrinsic apoptotic pathway, the so-called mitochondrial pathway [40]. The ratio between ROS production and ROS uptake is precisely controlled under physiological conditions. Unfortunately, dysregulation of this balance can lead to the initiation of various cellular responses, such as signaling pathways that are responsible for cell protection, initiation of coordinated activation of mitochondrial fission and autophagy to prevent spreading the damage to the neighboring mitochondria and cells [30]. ROS are discussed as signaling molecules and plays a role in communication with other cellular components. ROS such as hydrogen peroxide is diffusive, and therefore, influences the activity of proteins by regulating the oxidative state of one or more cysteine residues. This regulation is crucial in the control of many physiological processes [41]. Interestingly, the enhancement of ROS leads to the delay of aging and age-related diseases [42].

In this paper, we have also proven DNA condensation in HT-1080 cells after star polymer treatment, but we did not observe nuclear fragmentation (Figure 3A). Our findings are consistent with other authors [32,34] who confirmed that DNA condensation is one of the most characteristic features of apoptosis. An important hallmark of apoptotic cell is also internucleosomal DNA fragmentation [43]. HT-1080 cells treated with star polymer are DNA-deficient cells where discontinuity of DNA fragmentation may appear (Figure 3B). It may be altered by chromatin structure or involvement of different nucleases. Many factors such as Reactive Oxygen Species (ROS) treatment for 24 h [35], phosphatidylserine-Gold Nanoparticles [34] or dihydropyrimidinone-derived selenoesters [28] have been already reported as apoptosis inductors. Tone et al. (2007) [37] assessed that the population of nuclei undergoing apoptosis in vitro appears to undergo a reproducible program of nuclear condensation, suggesting the existence of an ordered biochemical pathway. Importantly, it is useful to assess other changes in cellular organelles and gene expression abnormalities, especially those characteristic of an apoptotic cell. 

Caspases are crucial players in apoptosis due to their ability to act as initiators and the executors of cell death [44]. We did not obtain any changes in caspase 8 expression but caspase 3 expression is slightly increased (Figure 4). This allows the assessment of treating cells with star polymer which does not activate mitochondrial apoptotic pathway. The intrinsic pathway is strictly regulated by the BCL-2 family of intracellular proteins. This protein regulates proapoptotic and antiapoptotic intrinsic pathways alternating mitochondrial outer membrane permeability [45]. Of note, the BCL-2 proteins are referred to as an “apoptotic switch”. Our results suggest that star polymer does not activate the intrinsic apoptotic pathway (Figure 4). Expression of the factors involved in this pathway remain unchanged and their expression is comparable to those in non-apoptotic cells. 

The correlation between ROS level and extrinsic-induced apoptosis has also been proven. An important regulator of the complex signaling network is a cytokine called TNF-α that promotes cell survival or death through apoptosis [46]. TNF-α can also cause caspase-independent necrosis (necroptosis). It involves ROS generation from either mitochondrial or non-mitochondrial pathways [38,47]. We proved that TNF expression is increased after cell treatment with star polymer (Figure 4).

Clearly, the JAK/STAT signaling pathway is fundamental in initiating apoptotic IFNs signals. Despite the importance of the JAK/STAT signaling pathway is still not well understood, it is clear that other signaling pathways are involved in the regulation of IFN-induced apoptosis. Nevertheless, huge progress has been made in terms of understanding how IFNs promote cell death. It provides ideal opportunities to design novel strategies to overcome resistance and enhance the therapeutic effects of IFNs [32]. It has been also demonstrated that IFNβ activates the TRAIL signaling pathway, leading to apoptosis in an autocrine manner [22]. Interestingly, there are findings that have revealed that IL-1β and TNFα induce apoptosis by FasL activation. IL-1β and TNFα differentially influence NF-κB activity leading to differential upregulation of target genes, which may contribute to the distinct effects on cell viability [46].

Apoptosis inducers’ action may be cell line dependent. Despite how different cell types may die in apoptotic pathways, its mechanism can be diversified. In our studies, we also tested the toxicity of star polymer for human bladder cancer cell lines 5637 and T24 and for various normal cells lines: PDF, HMEC and BdEC cells. We observed a similar trend to that found in HT-1080 cells. Again, the cell viability was lower after 24 h compared to 6 h of cells treatment with star polymer (Figure 5A) and their morphology has been changed (Figure 5B). We also visualized shrunken nuclei and proved that these cells undergo an early stage of apoptosis (Figure 6A,B).

We demonstrate the highlighting potential of star polymers as an innovative tool for anticancer therapy. These nanoparticles can be used as apoptosis triggers in various cell lines. Our findings revealed that they are able to perform apoptosis induction in HT-1080 cells, human bladder cancer cell lines 5637 and T24 and human dermal fibroblasts (PDF). Importantly, used STAR showed toxic effect for cancer cells but this effect was lower for normal cell lines: HMEC cells and BdEC cells. However, the study of the apoptosis phenomenon remains a challenge that needs to be addressed in future research. Knowing that defects in apoptosis are associated with many types of diseases such as autoimmune diseases, neurodegenerative diseases, heart diseases, and cancer, the ability to target apoptosis in specific cells could be a potential drug candidate.

## Data Availability

Not applicable.

## Supplementary Materials

The following supporting information can be downloaded at: https://www.mdpi.com/article/10.3390/biom12050608/s1, Table S1: Dh and zeta potential of the star polymer with 28 poly(*N,N*′-dimethylaminoethyl methacrylate) arms; Table S2: Primers used in qRT-PCR analysis; Figure S1: Kinetics of star polymer action on Primary Mammary Epithelial Cells (HMECs) and Primary Bladder Epithelial Cells (BdECs). Cytotoxicity dynamics of star polymer for HMECs and BdECs cell lines after 6 and 24 h of treatment are presented.
