# Supplementary material for: Star Polymers as Non-Viral Carriers for Apoptosis Induction
Source: Biomolecules. 2022 Apr 19;12(5):608. doi: 10.3390/biom12050608 (PMC9139127; doi:10.3390/biom12050608)
Supplement: Supplementary file 1 [file biomolecules-12-00608-s001.zip › biomolecules-1658324-supplementary.pdf]

Table S1. Hydrodynamic diameter Dh and zeta potential values of the star polymer with 28 poly(*N,N'*-dimethylaminoethyl methacrylate) arms.

Star polymer	Zeta potential in aqueous solution at pH=7.4 (mV)	Zeta potential in DMEM at pH=7.4 (mV)	D_h in acetone	D_h in aqueous solution	D_h in DMEM
STAR	50 mV	18.6 mV	18.8 nm	38 nm, 48 nm (two populations)	39 nm

The smallest size of star polymer has been observed in acetone which is good solvent for the core and arms of the star. In other solvents, there is a tendency to a slight aggregation which results from the amphiphilic nature of the polymer (the core is hydrophobic and the arms are hydrophilic).

Regardless, the small sizes of the star polymers in all studied solvents are still acceptable for biological and medical applications.

Table S2. Primers used in qRT-PCR analysis.

Gene	Forward primer (5'-3')	Reverse primer (5'-3')
**Bax**	CTGGACATTGGACTTCCTC	AAGATGGTCACGGTCTGCCA
**Bcl-2**	CCCTGTGGATGACTGAGTACC	GACAGCCAGGAGAAATCAAACAG
**Cas 3**	TGGTTCATCCAGTCGCTTTG	CATTCTGTTGCCACCTTTCG
**Cas 8**	CTGCTGGGGATGGCCACTG	TCGCCTCGAGGACACGCTCTC
**Cas 9**	CGAACTAACAGGCAAGCAGC	CGAACTAACAGGCAAGCAGC
**HO-1**	GTGCTCAAAAAGATTGCCCA	AAAGCTGAGTGTAAGGACCC
**IL-1β**	CTTATCCCATAGCCAGGAAAC	GGTGGAATTGAATGAAACAAGAA
**IL-6**	TGAACCTTCCAAAGATGGCT	CTGGCTTGTTCCTCACTACT
**INTα**	AGGTTGTCAGAGCAGAAATCA	TATAGCAGGGGTGAGAGTCTT
**INTβ**	TCCATGAGCTACAACTTGCT	CCTTGAGGCAGTATTCAAGC
**INTγ**	GAGTGTGGAGACCATCAAGGA	CTGTTTTAGCTGCTGGCGAC
**TNF**	CCAACTGTCACTCATTGCTG	CTCGCCACTGAATAGTAGGG
**GAPDH**	GAGTCAACGGATTTGGTCGTA	GCCCCACTTGATTTTGGAG

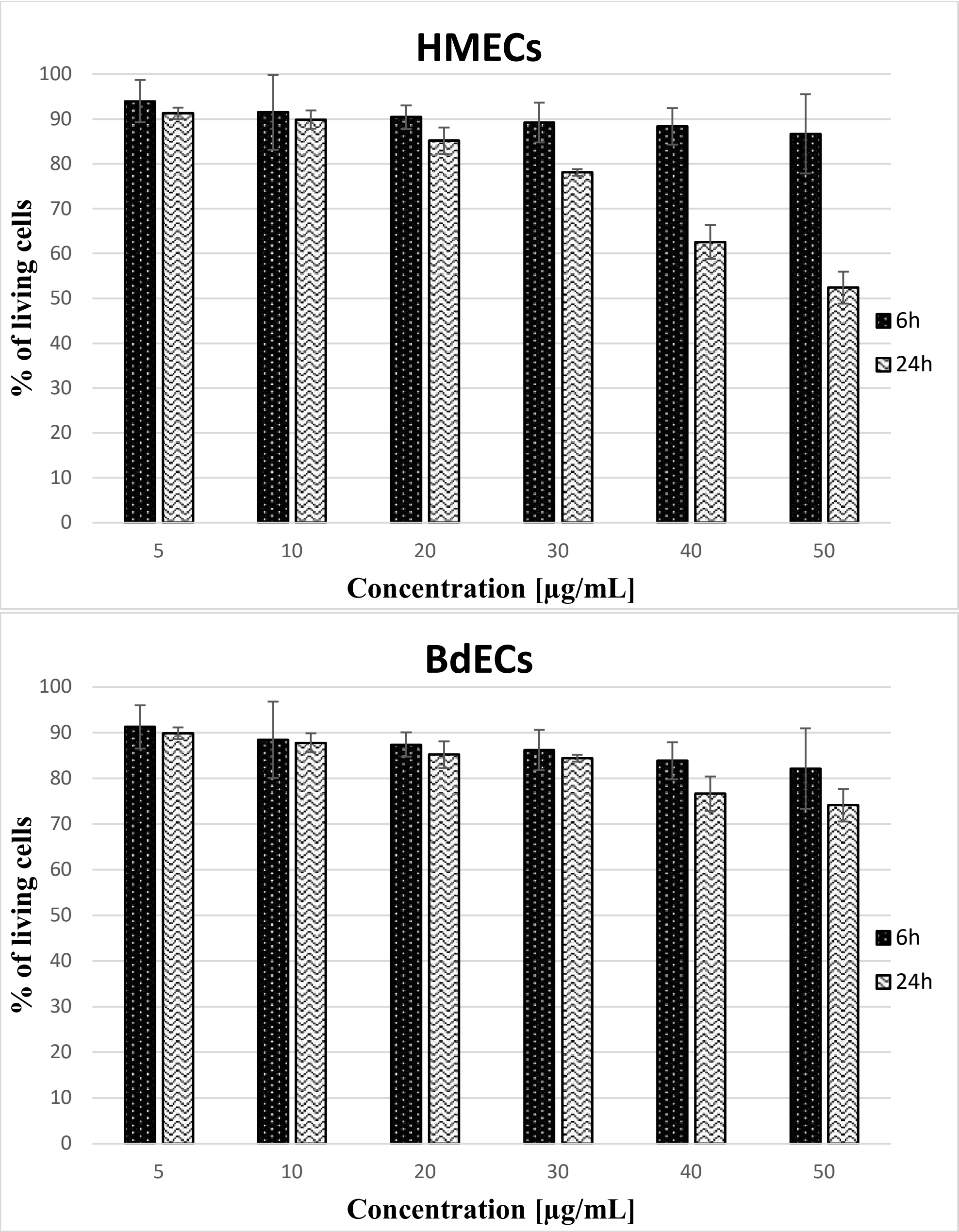

Figure S1. Kinetics of star polymer action on Primary Mammary Epithelial Cells (HMECs) and Primary Bladder Epithelial Cells (BdECs). Cytotoxicity dynamics of star polymer for HMECs and BdECs cell lines after 6 and 24h of treatment are presented.
